# Correction: Metabolomics approach for predicting stomach and colon contents in dead *Arctocephalus pusillus pusillus*, *Arctocephalus tropicalis*, *Lobodon carcinophaga* and *Ommatophoca rossii* from sub-Antarctic region

**DOI:** 10.1371/journal.pone.0302976

**Published:** 2024-04-30

**Authors:** The PLOS ONE Staff

## Notice of Republication

This article was republished on April 17, 2024, to correct errors in the author names that were overlooked during the publication process. The publisher apologizes for these errors. Please download the article again to view the corrected version. Both the originally published, uncorrected article and the republished, corrected version are provided here for reference.

## Supporting information

S1 File Originally published, uncorrected article. (PDF)

S2 File Republished, corrected article. (PDF)
