# Supplementary material for: Metabolomics approach for predicting stomach and colon contents in dead Arctocephalus pusillus pusillus, Arctocephalus tropicalis, Lobodon carcinophaga and Ommatophoca rossii from sub-Antarctic region
Source: PLoS One. 2024 Apr 1;19(4):e0300319. doi: 10.1371/journal.pone.0300319 (PMC10984408; doi:10.1371/journal.pone.0300319)
Supplement: S1 File — (PDF) [file pone.0300319.s001.pdf]

S1 File. Typical examples of the metabolite's spectra from the stomach and colon samples of *Arctocephalus pusillus, Arctocephalus tropicalis, Lobodon carcinophaga* and *Ommatophoca rossii.*

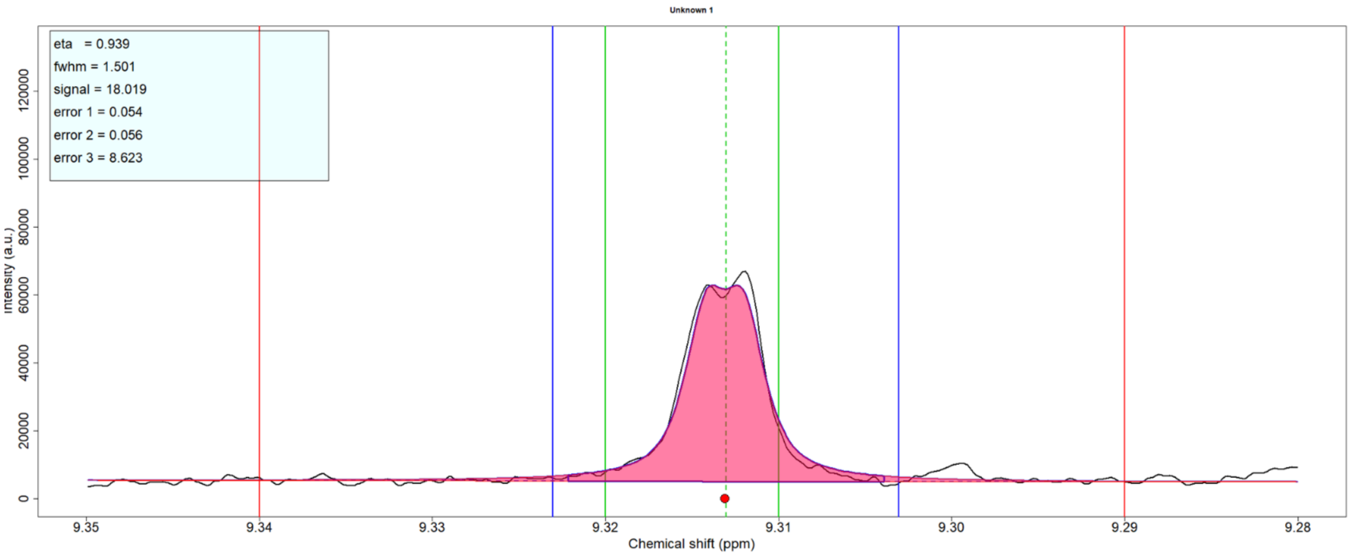

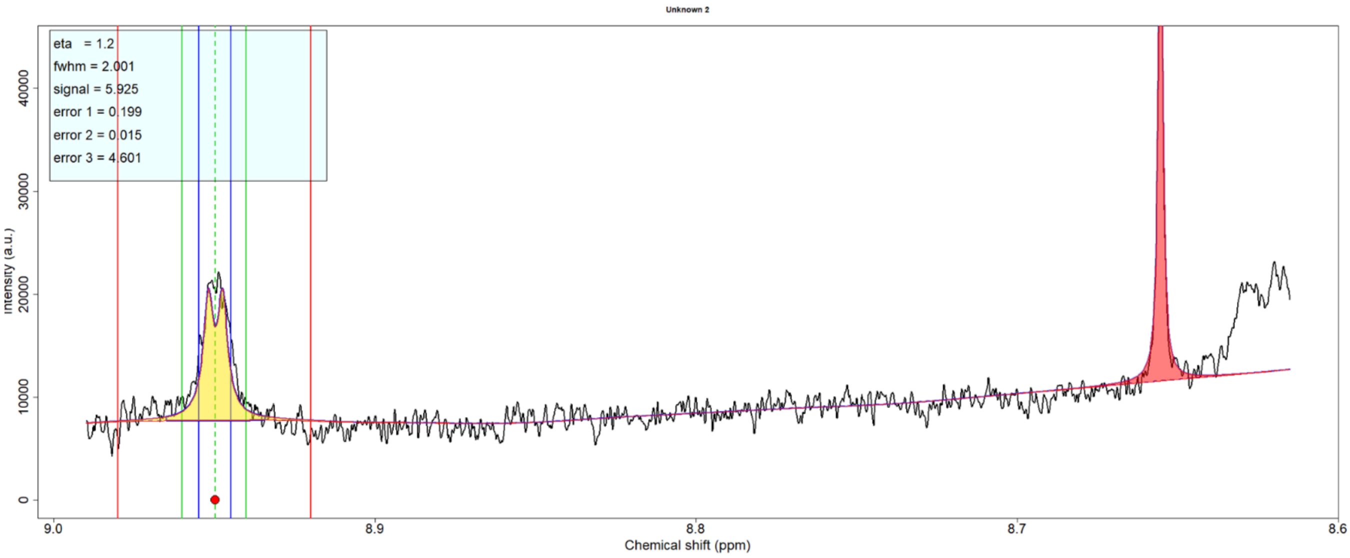

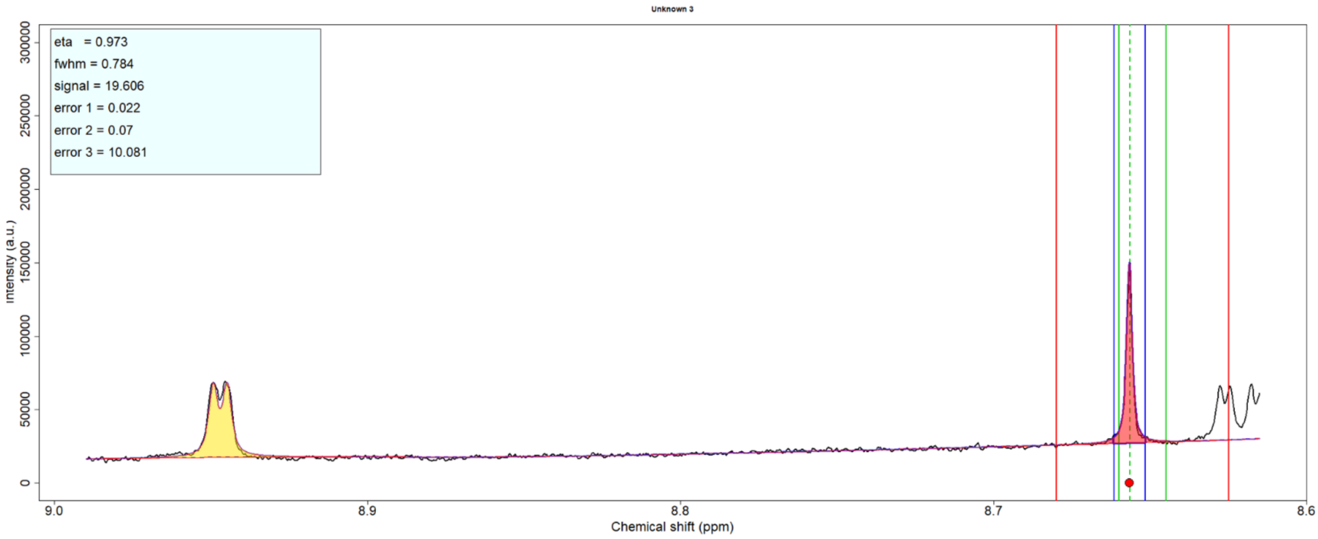
Unknown 3
eta = 0.973
fwhm = 0.784
signal = 19.606
error 1 = 0.022
error 2 = 0.07
error 3 = 10.081
Intensity (a.u.)
0
50000
100000
150000
200000
250000
300000
9.0
8.9
8.8
8.7
8.6
Chemical shift (ppm)

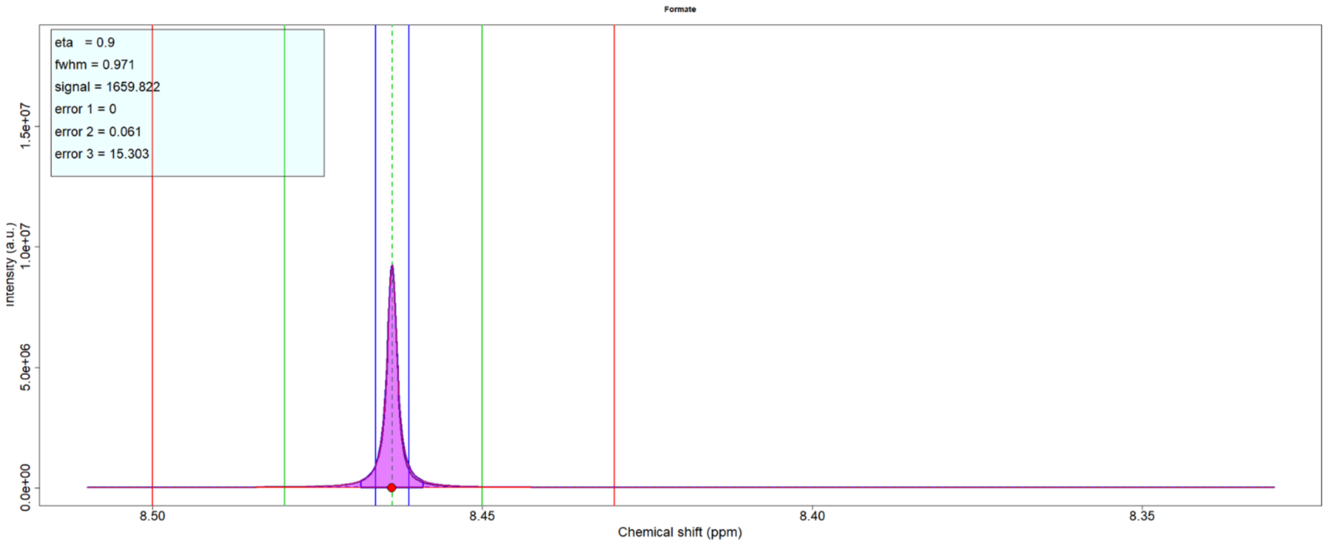
Formate
eta = 0.9
fwhm = 0.971
signal = 1659.822
error 1 = 0
error 2 = 0.061
error 3 = 15.303
Intensity (a.u.)
0.0e+00
5.0e+06
1.0e+07
1.5e+07
8.50
8.45
8.40
8.35
Chemical shift (ppm)

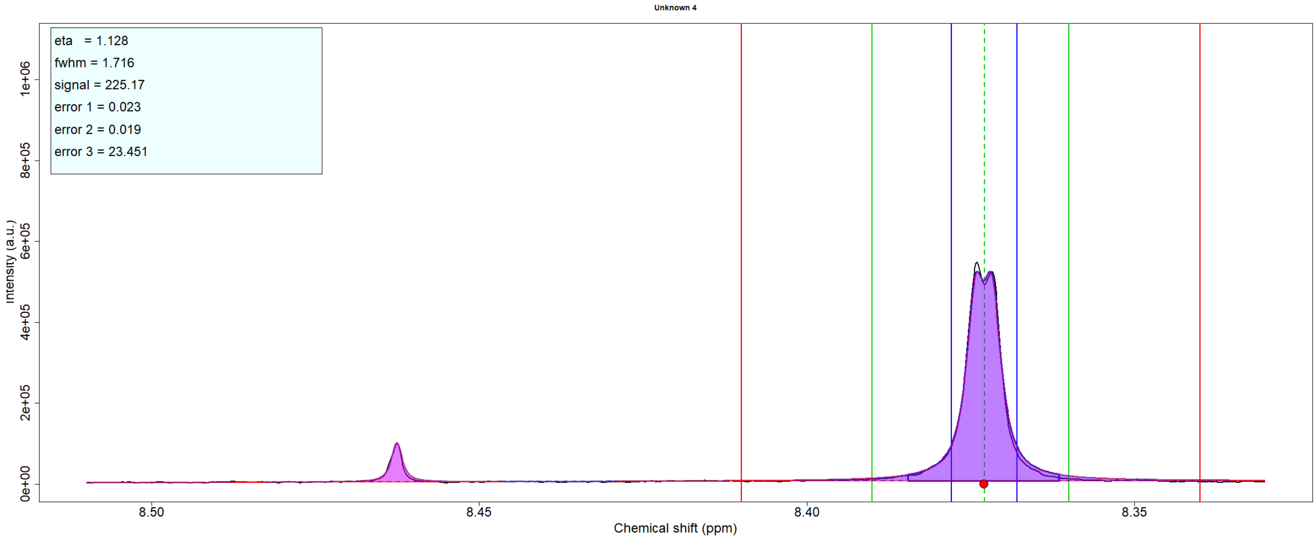
Unknown 4
eta = 1.128
fwhm = 1.716
signal = 225.17
error 1 = 0.023
error 2 = 0.019
error 3 = 23.451
Intensity (a.u.)
0e+00
2e+05
4e+05
6e+05
8e+05
1e+06
8.50
8.45
8.40
8.35
Chemical shift (ppm)

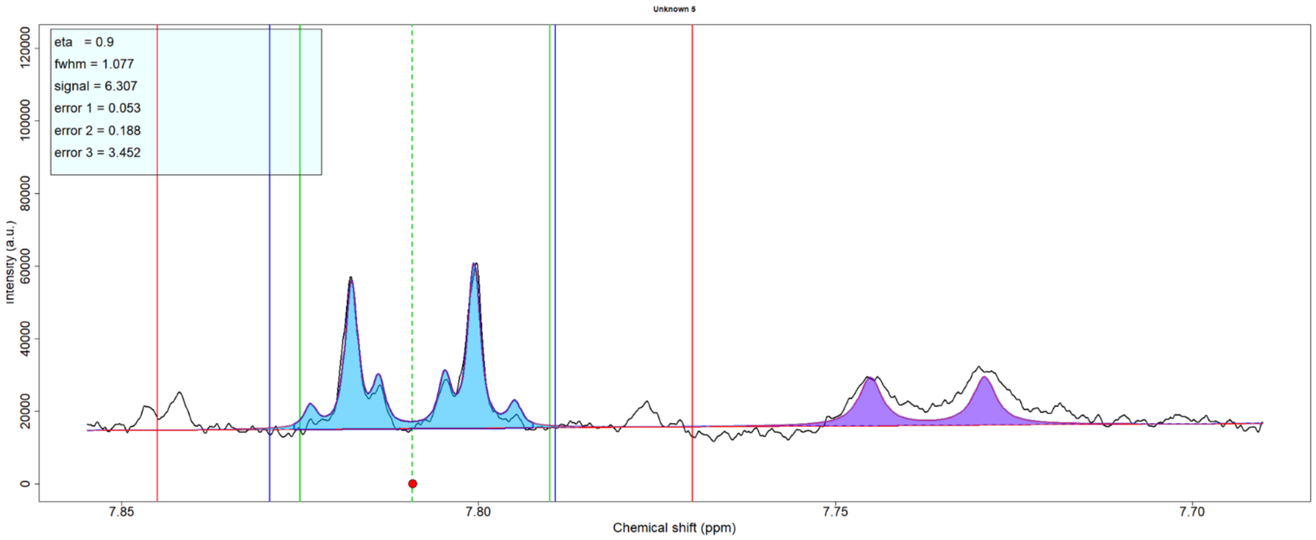
Unknown 5
eta = 0.9
fwhm = 1.077
signal = 6.307
error 1 = 0.053
error 2 = 0.188
error 3 = 3.452
intensity (a.u.)
Chemical shift (ppm)

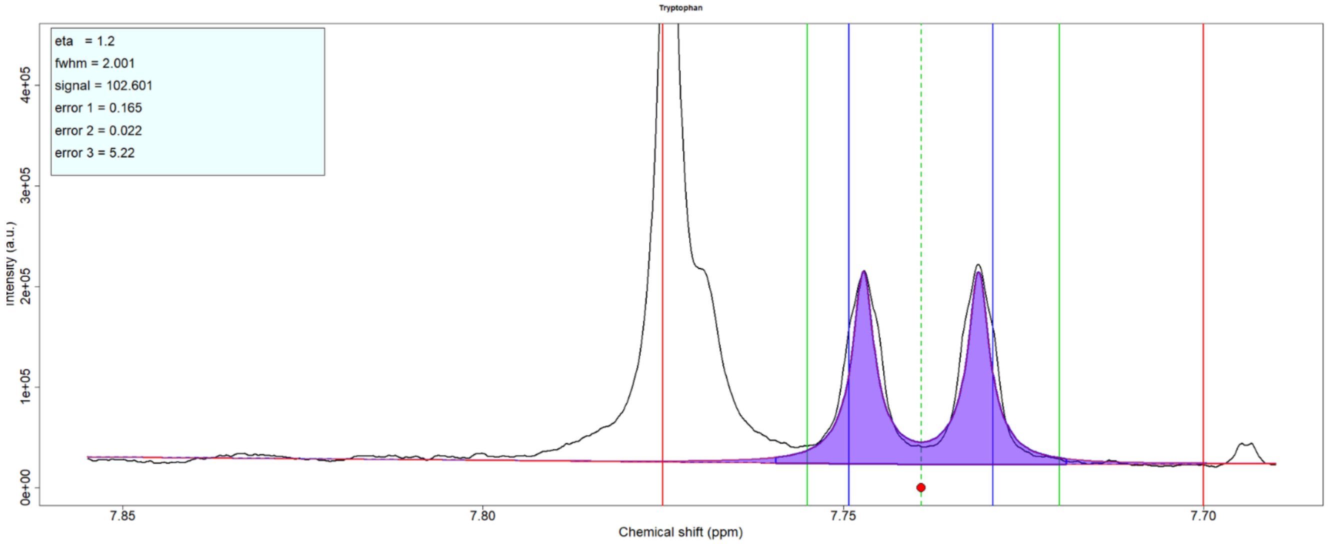
Tryptophan
eta = 1.2
fwhm = 2.001
signal = 102.601
error 1 = 0.165
error 2 = 0.022
error 3 = 5.22
intensity (a.u.)
Chemical shift (ppm)

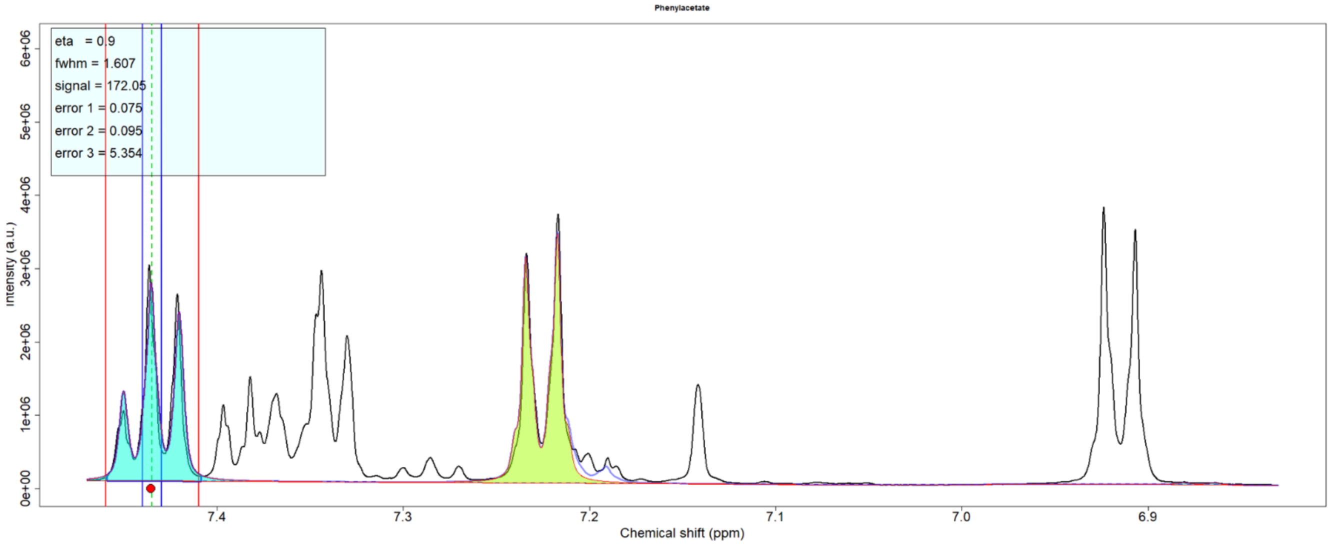
Phenylacetate
eta = 0.9
fwhm = 1.607
signal = 172.05
error 1 = 0.075
error 2 = 0.095
error 3 = 5.354
intensity (a.u.)
Chemical shift (ppm)

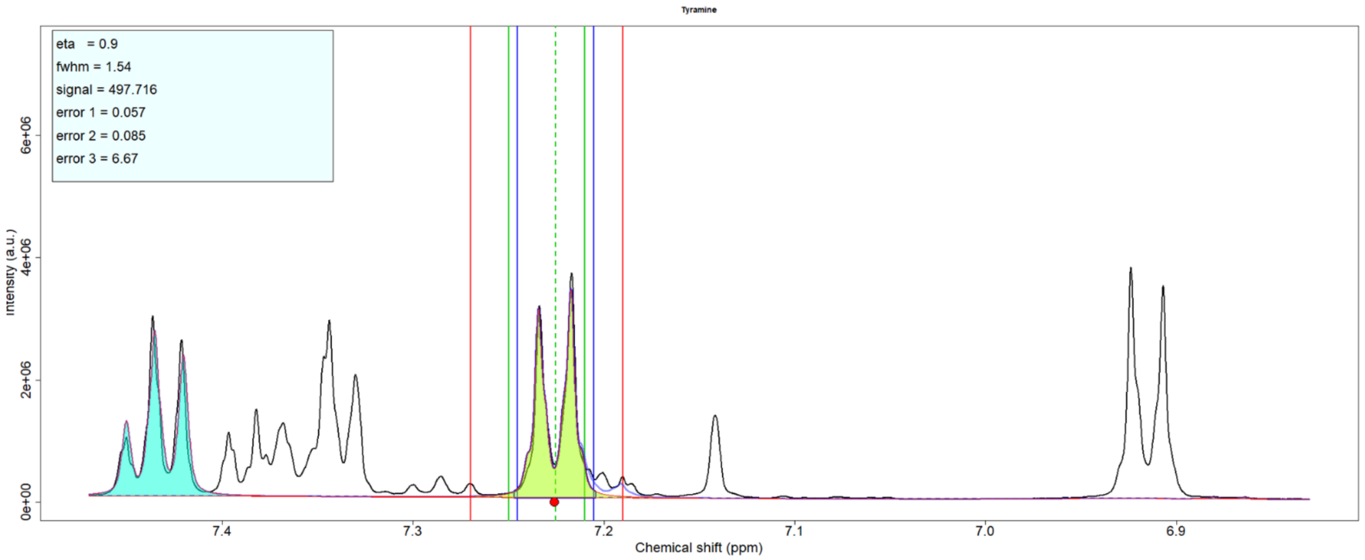
Tyramine
eta = 0.9
fwhm = 1.54
signal = 497.716
error 1 = 0.057
error 2 = 0.085
error 3 = 6.67
intensity (a.u.)
0e+00
2e+06
4e+06
6e+06
7.4
7.3
7.2
7.1
7.0
6.9
Chemical shift (ppm)

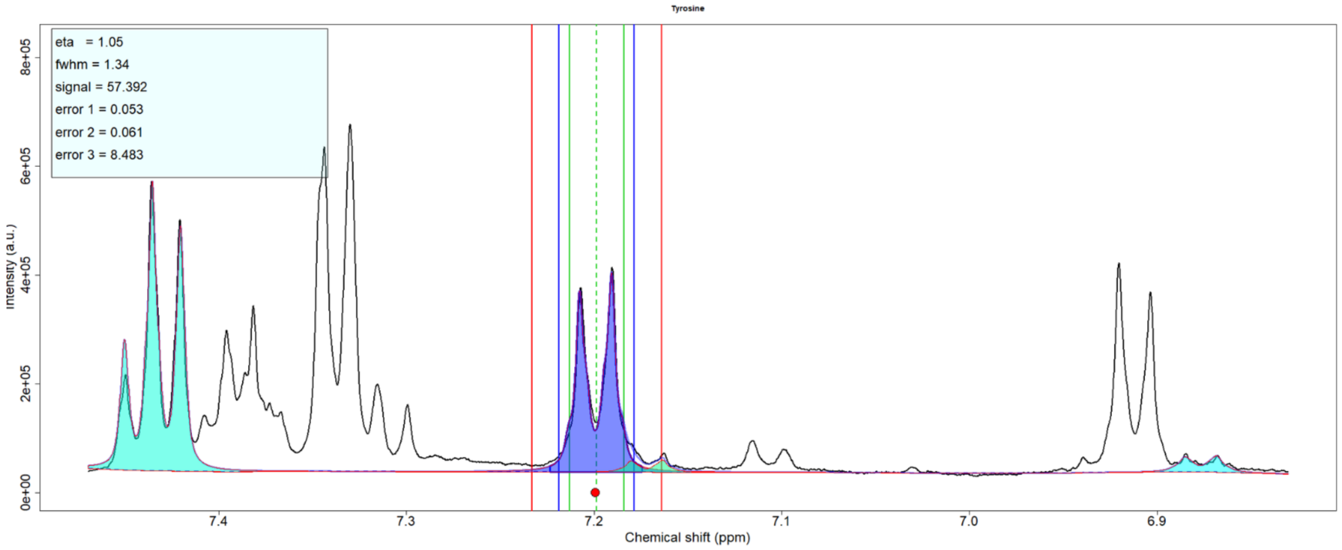
Tyrosine
eta = 1.05
fwhm = 1.34
signal = 57.392
error 1 = 0.053
error 2 = 0.061
error 3 = 8.483
intensity (a.u.)
0e+00
2e+05
4e+05
6e+05
8e+05
7.4
7.3
7.2
7.1
7.0
6.9
Chemical shift (ppm)

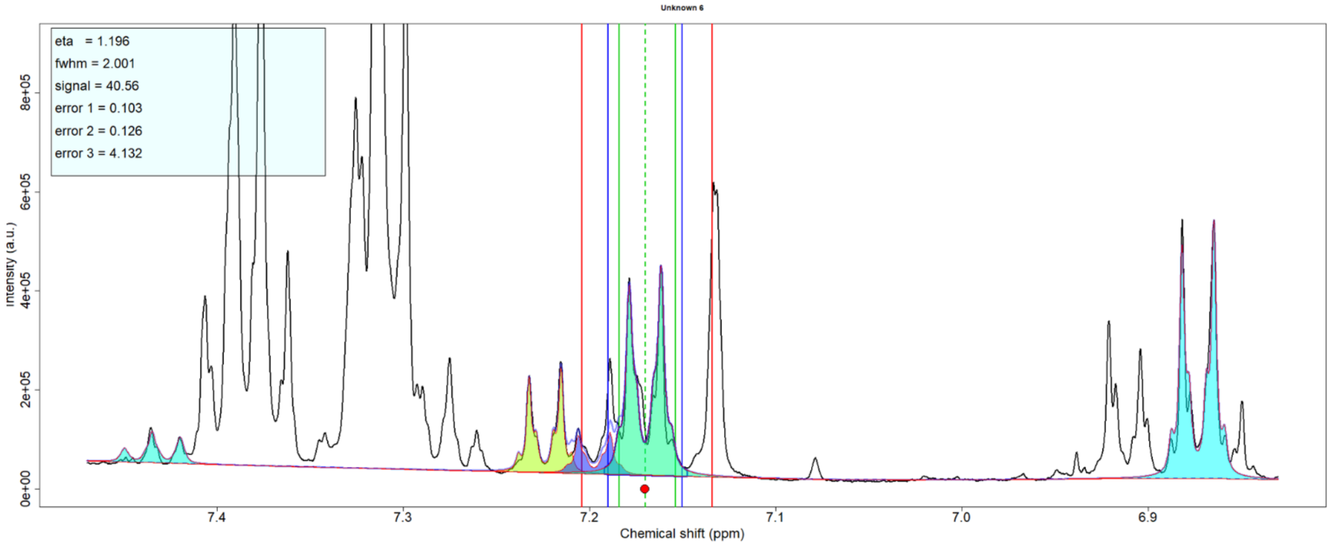
Unknown 6
eta = 1.196
fwhm = 2.001
signal = 40.56
error 1 = 0.103
error 2 = 0.126
error 3 = 4.132
intensity (a.u.)
0e+00
2e+05
4e+05
6e+05
8e+05
7.4
7.3
7.2
7.1
7.0
6.9
Chemical shift (ppm)

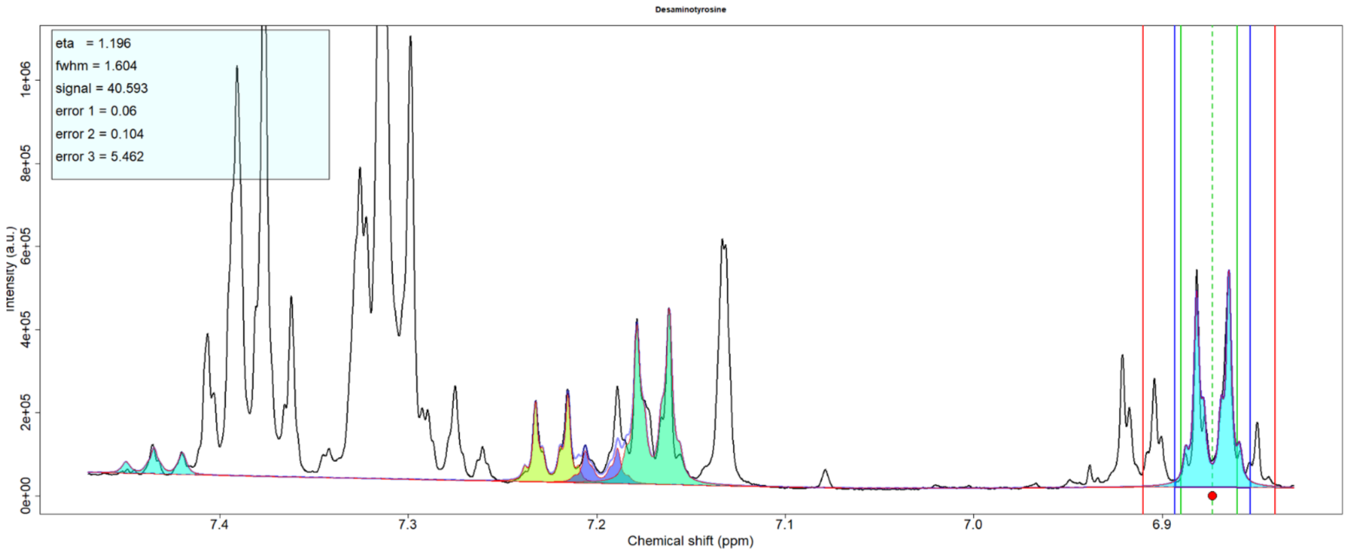
Desaminotyrosine
eta = 1.196
fwhm = 1.604
signal = 40.593
error 1 = 0.06
error 2 = 0.104
error 3 = 5.462
intensity (a.u.)
Chemical shift (ppm)

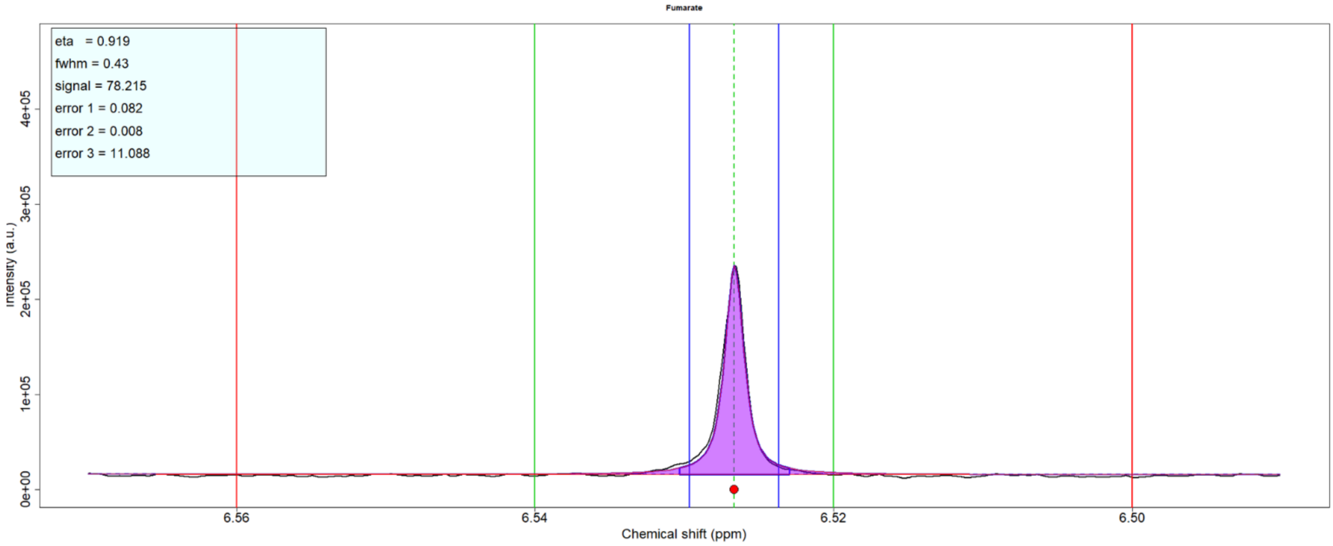
Fumarate
eta = 0.919
fwhm = 0.43
signal = 78.215
error 1 = 0.082
error 2 = 0.008
error 3 = 11.088
intensity (a.u.)
Chemical shift (ppm)

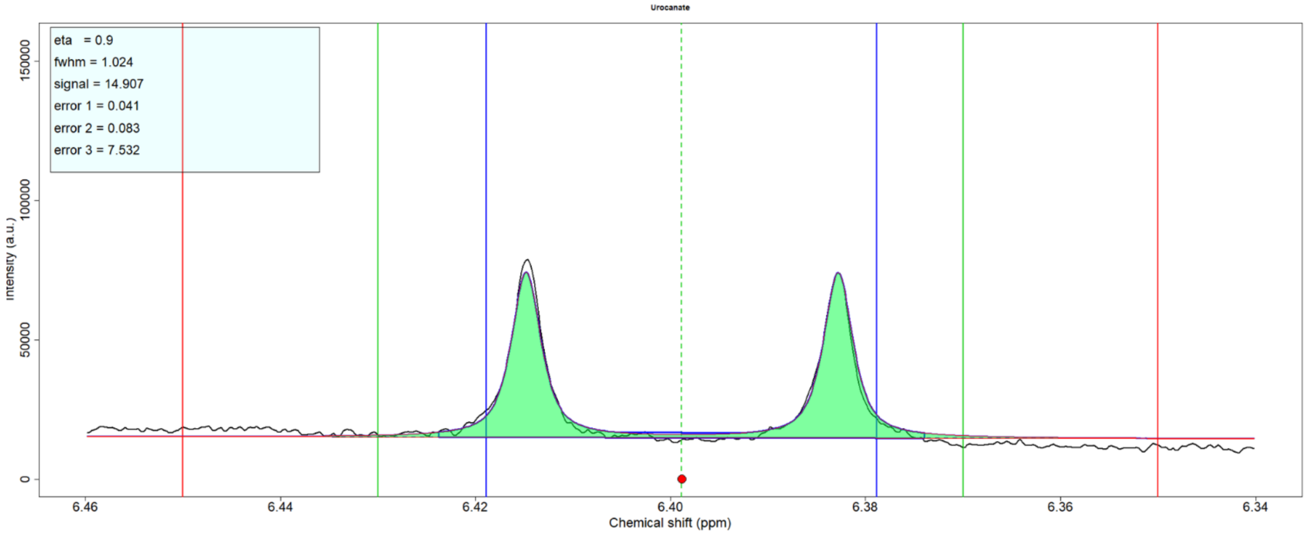
Urocanate
eta = 0.9
fwhm = 1.024
signal = 14.907
error 1 = 0.041
error 2 = 0.083
error 3 = 7.532
intensity (a.u.)
Chemical shift (ppm)

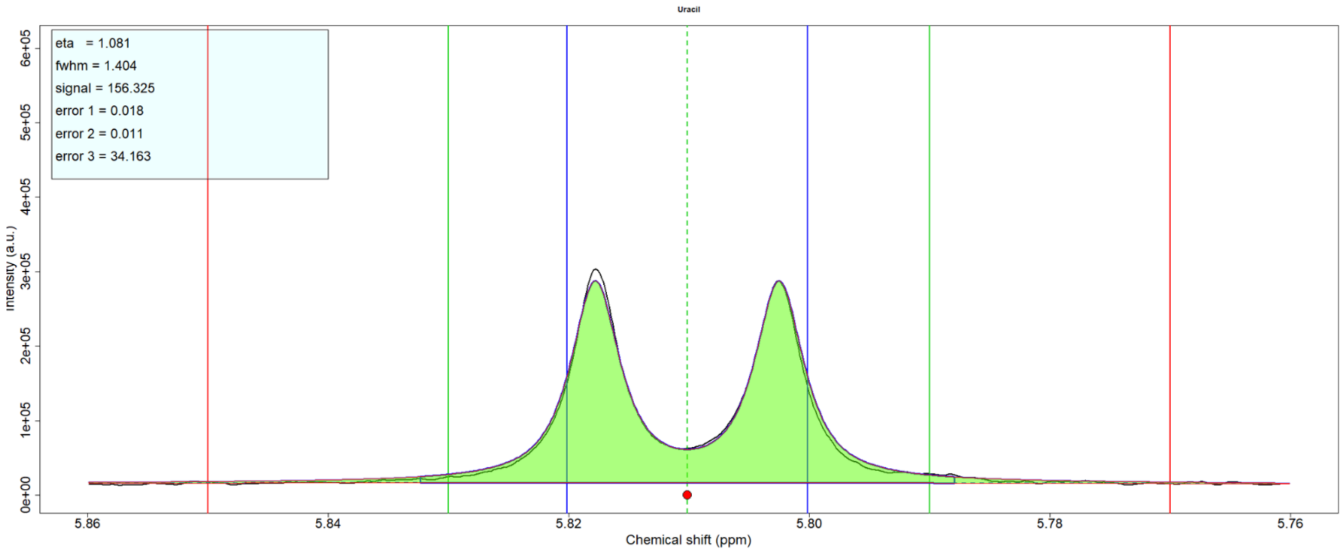
Uracil
eta = 1.081
fwhm = 1.404
signal = 156.325
error 1 = 0.018
error 2 = 0.011
error 3 = 34.163
Intensity (a.u.)
0e+00
1e+05
2e+05
3e+05
4e+05
5e+05
6e+05
5.86
5.84
5.82
5.80
5.78
5.76
Chemical shift (ppm)

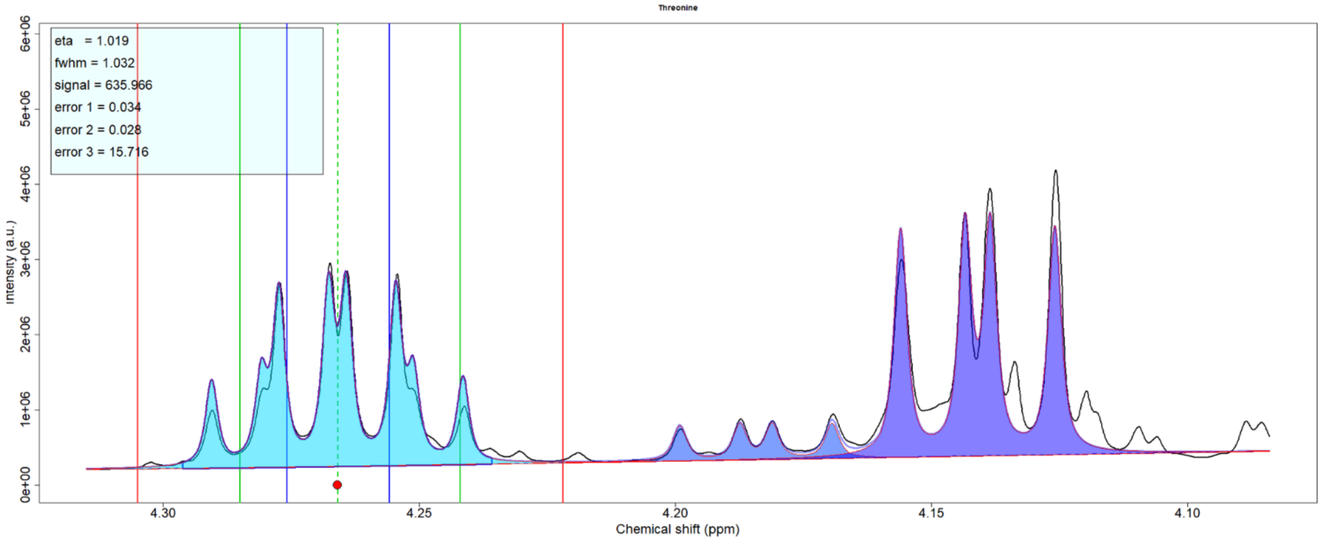
Threonine
eta = 1.019
fwhm = 1.032
signal = 635.966
error 1 = 0.034
error 2 = 0.028
error 3 = 15.716
Intensity (a.u.)
0e+00
1e+06
2e+06
3e+06
4e+06
5e+06
6e+06
4.30
4.25
4.20
4.15
4.10
Chemical shift (ppm)

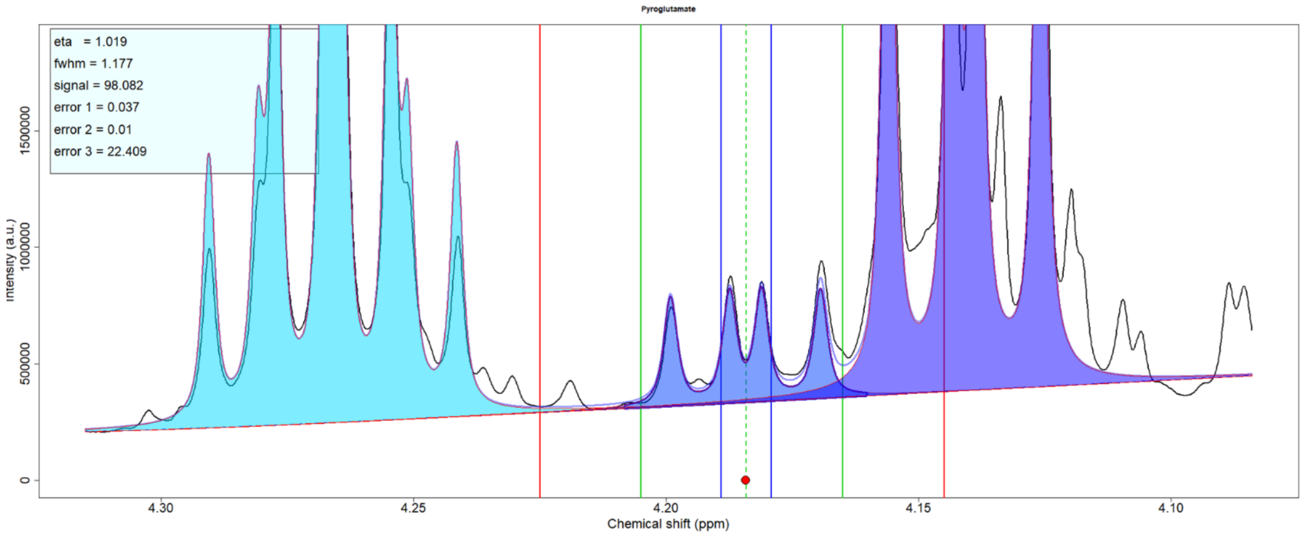
Pyroglutamate
eta = 1.019
fwhm = 1.177
signal = 98.082
error 1 = 0.037
error 2 = 0.01
error 3 = 22.409
Intensity (a.u.)
0
500000
1000000
1500000
4.30
4.25
4.20
4.15
4.10
Chemical shift (ppm)

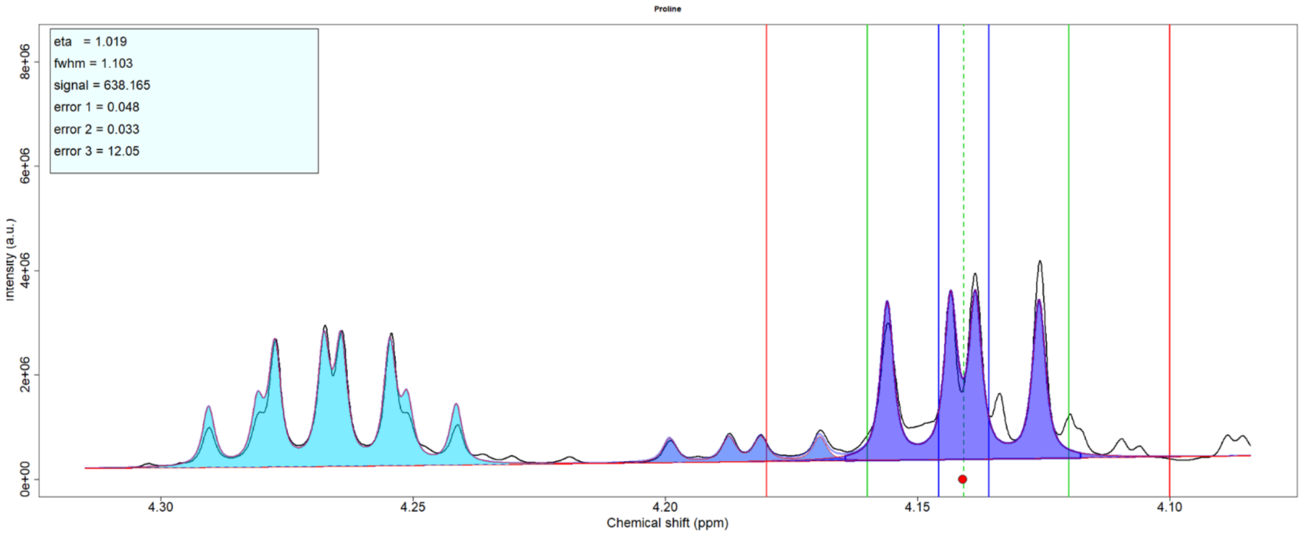
Proline
eta = 1.019
fwhm = 1.103
signal = 638.165
error 1 = 0.048
error 2 = 0.033
error 3 = 12.05
intensity (a.u.)
0e+00
2e+06
4e+06
6e+06
8e+06
4.30
4.25
4.20
4.15
4.10
Chemical shift (ppm)

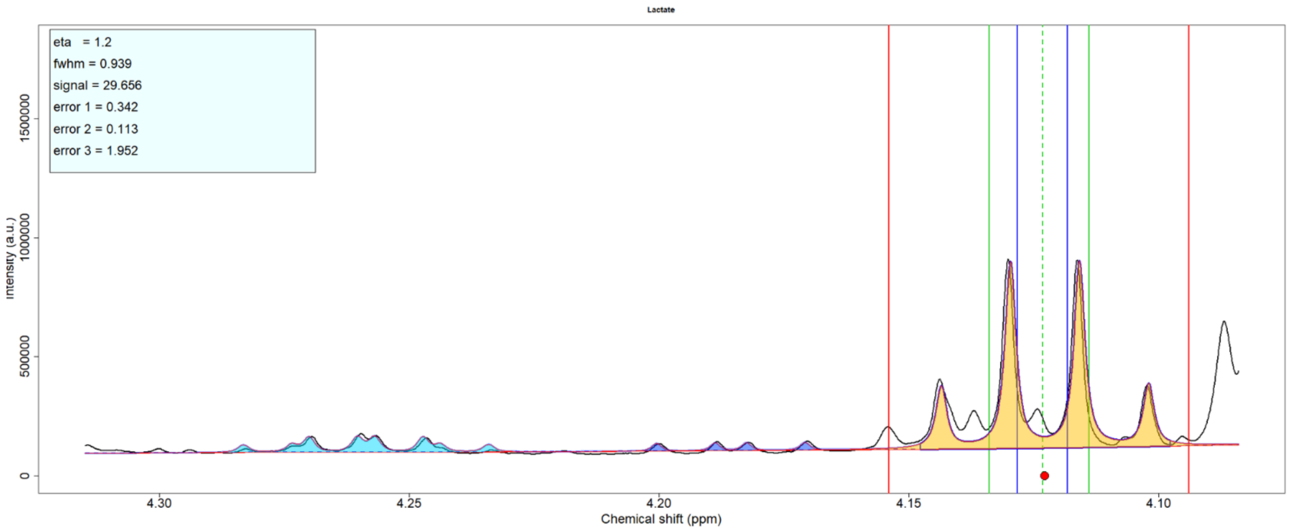
Lactate
eta = 1.2
fwhm = 0.939
signal = 29.656
error 1 = 0.342
error 2 = 0.113
error 3 = 1.952
intensity (a.u.)
0
500000
1000000
1500000
4.30
4.25
4.20
4.15
4.10
Chemical shift (ppm)

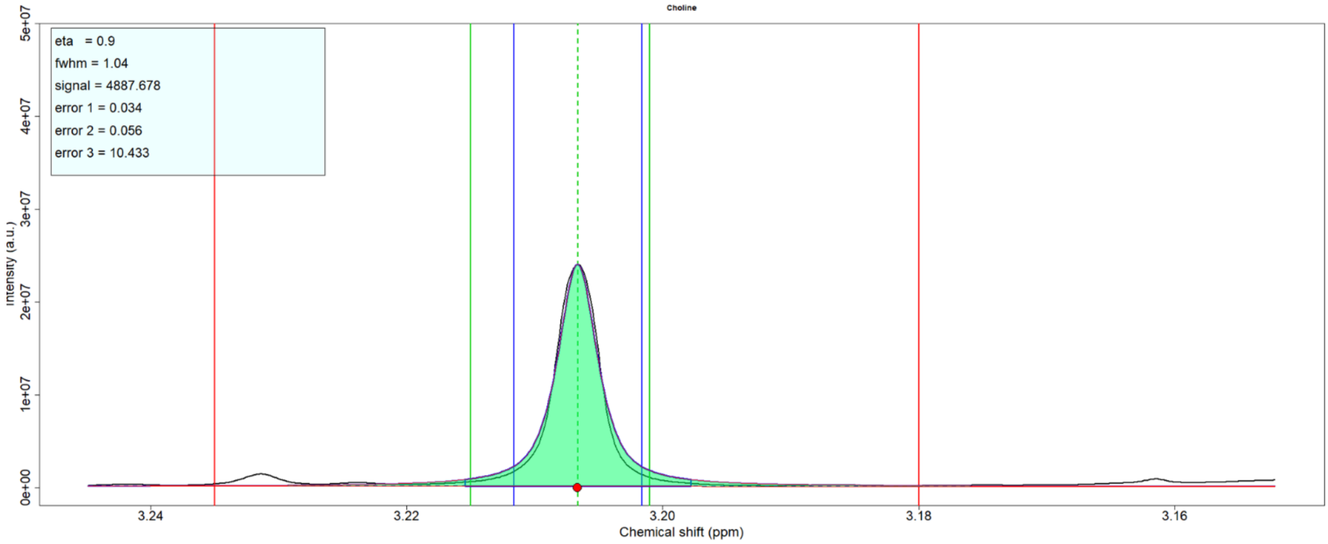
Choline
eta = 0.9
fwhm = 1.04
signal = 4887.678
error 1 = 0.034
error 2 = 0.056
error 3 = 10.433
intensity (a.u.)
0e+00
1e+07
2e+07
3e+07
4e+07
5e+07
3.24
3.22
3.20
3.18
3.16
Chemical shift (ppm)

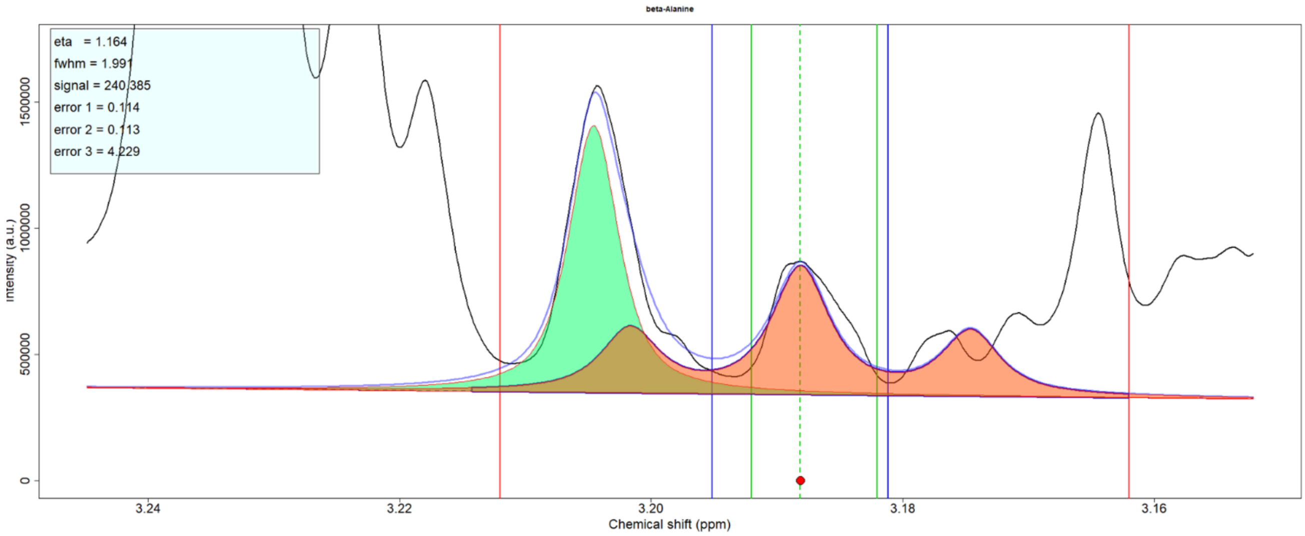
beta-Alanine
eta = 1.164
fwhm = 1.991
signal = 240.385
error 1 = 0.114
error 2 = 0.113
error 3 = 4.229
Intensity (a.u.)
Chemical shift (ppm)

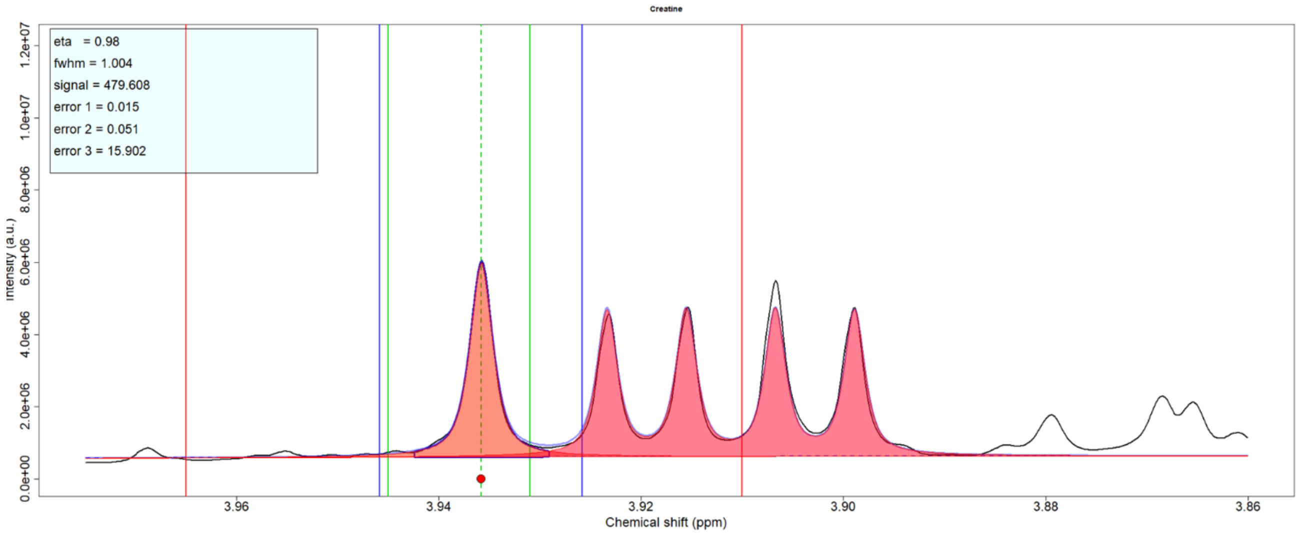
Creatine
eta = 0.98
fwhm = 1.004
signal = 479.608
error 1 = 0.015
error 2 = 0.051
error 3 = 15.902
Intensity (a.u.)
Chemical shift (ppm)

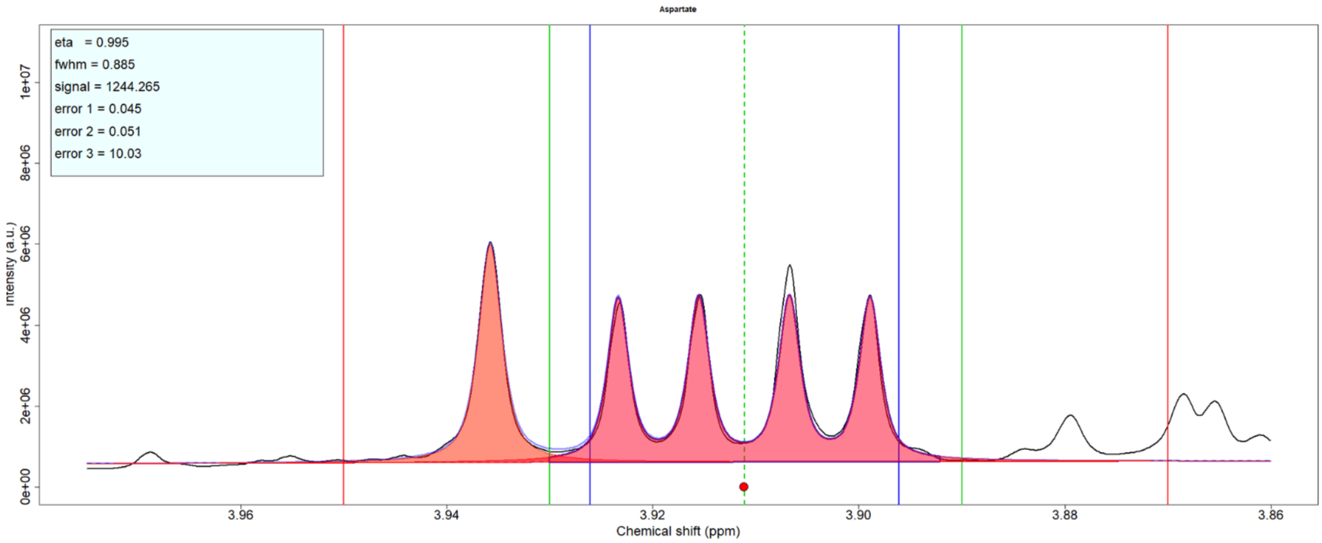
Aspartate
eta = 0.995
fwhm = 0.885
signal = 1244.265
error 1 = 0.045
error 2 = 0.051
error 3 = 10.03
Intensity (a.u.)
Chemical shift (ppm)

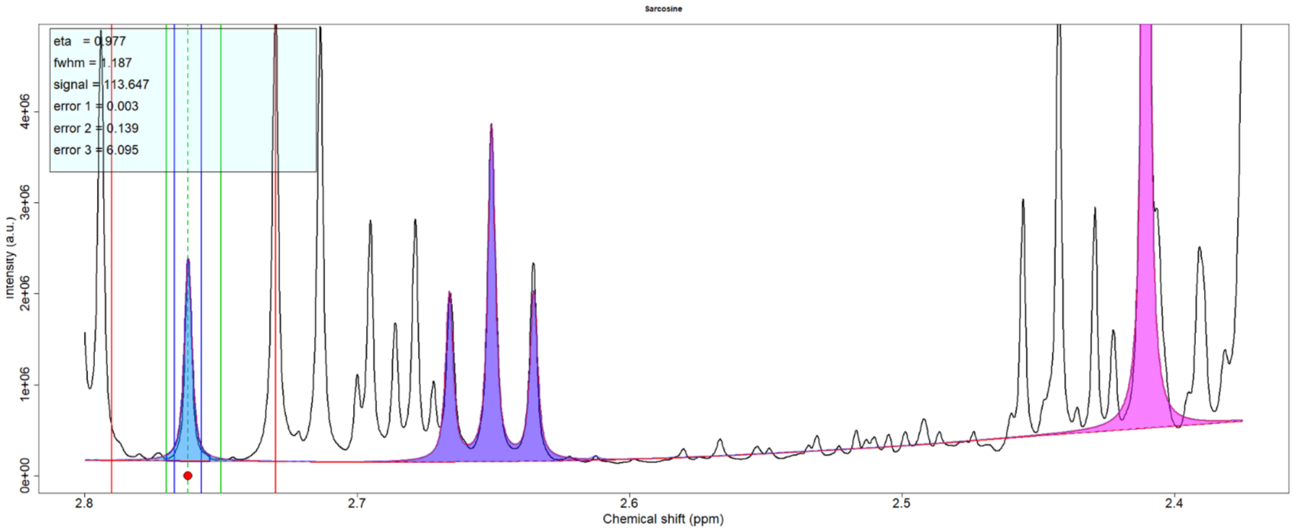
Sarcosine
eta = 0.977
fwhm = 1.187
signal = 113.647
error 1 = 0.003
error 2 = 0.139
error 3 = 6.095
intensity (a.u.)
0e+00
1e+06
2e+06
3e+06
4e+06
2.8
2.7
2.6
2.5
2.4
Chemical shift (ppm)

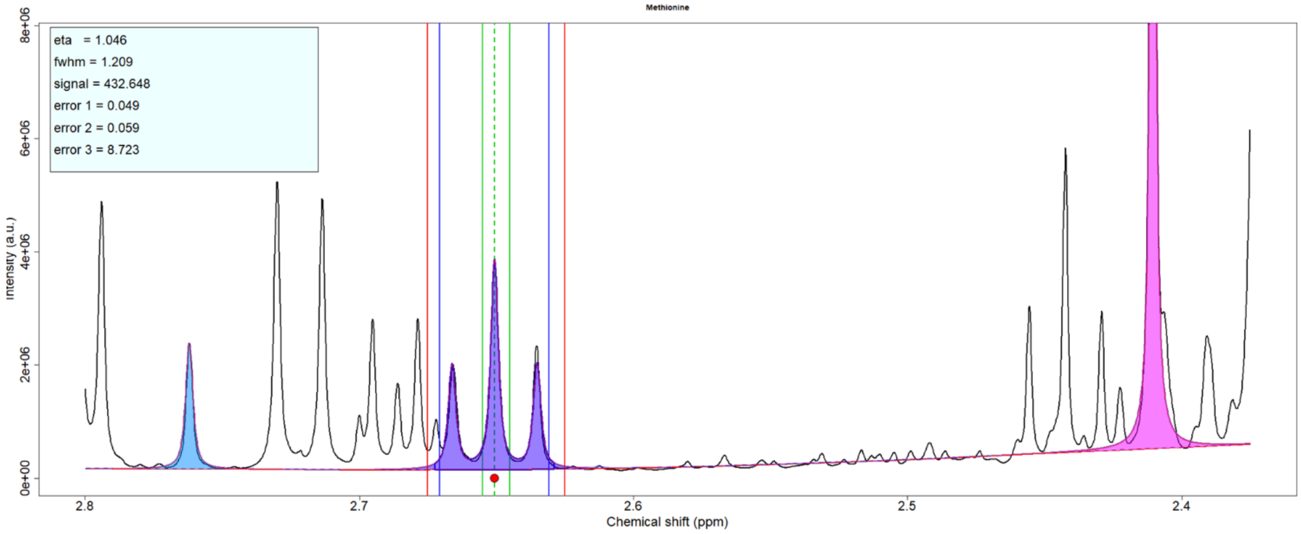
Methionine
eta = 1.046
fwhm = 1.209
signal = 432.648
error 1 = 0.049
error 2 = 0.059
error 3 = 8.723
intensity (a.u.)
0e+00
2e+06
4e+06
6e+06
8e+06
2.8
2.7
2.6
2.5
2.4
Chemical shift (ppm)

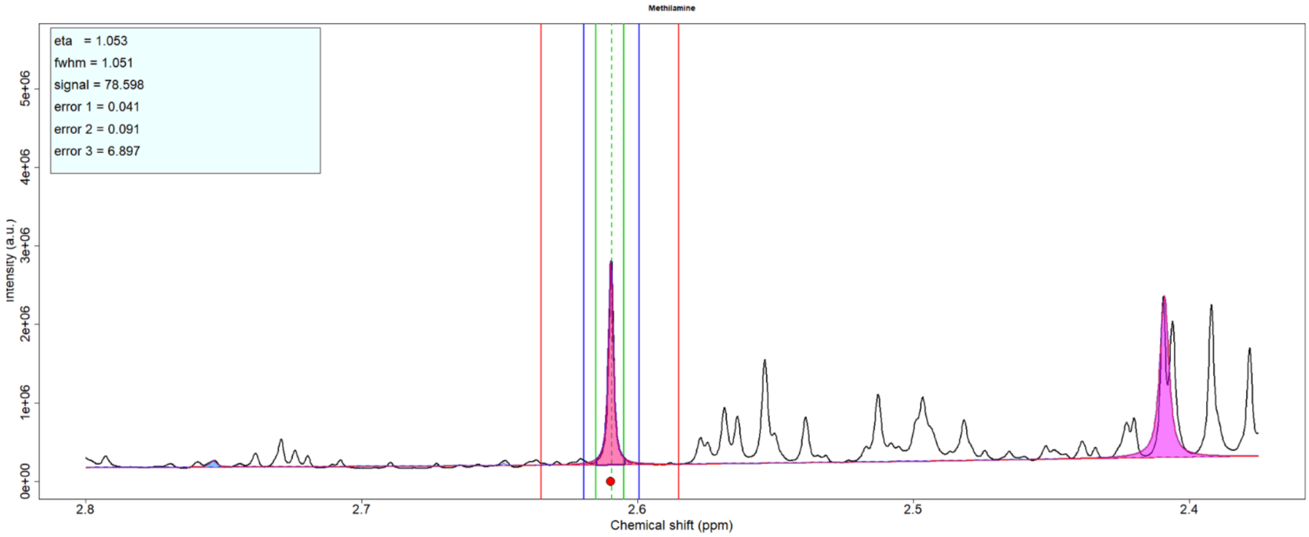
Methilamine
eta = 1.053
fwhm = 1.051
signal = 78.598
error 1 = 0.041
error 2 = 0.091
error 3 = 6.897
intensity (a.u.)
0e+00
1e+06
2e+06
3e+06
4e+06
5e+06
2.8
2.7
2.6
2.5
2.4
Chemical shift (ppm)

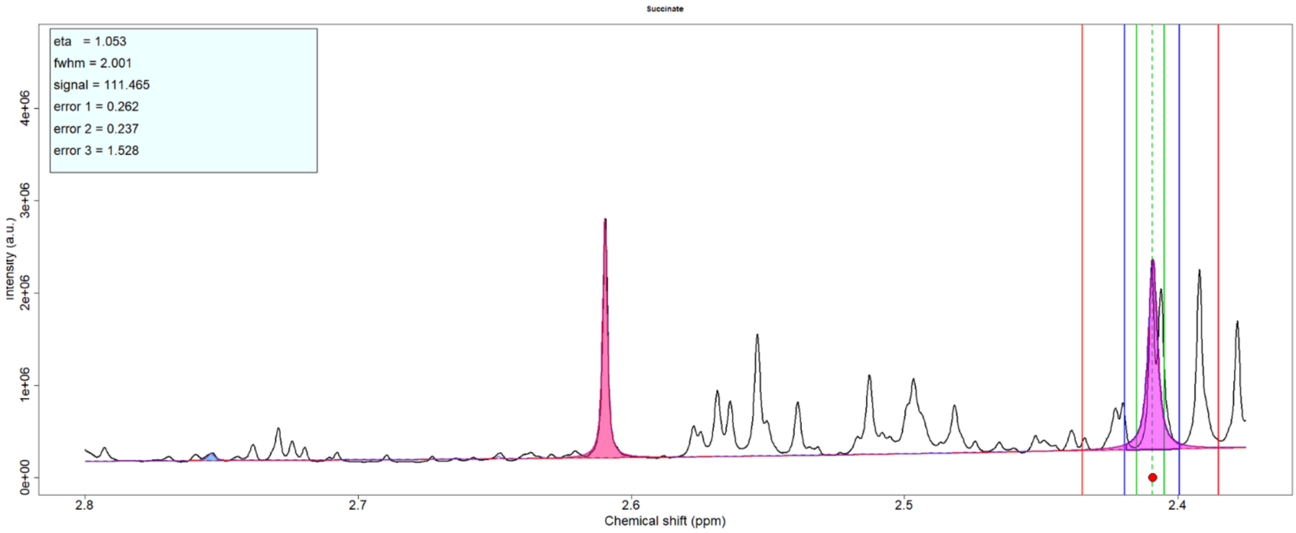
Succinate
eta = 1.053
fwhm = 2.001
signal = 111.465
error 1 = 0.262
error 2 = 0.237
error 3 = 1.528
Intensity (a.u.)
Chemical shift (ppm)

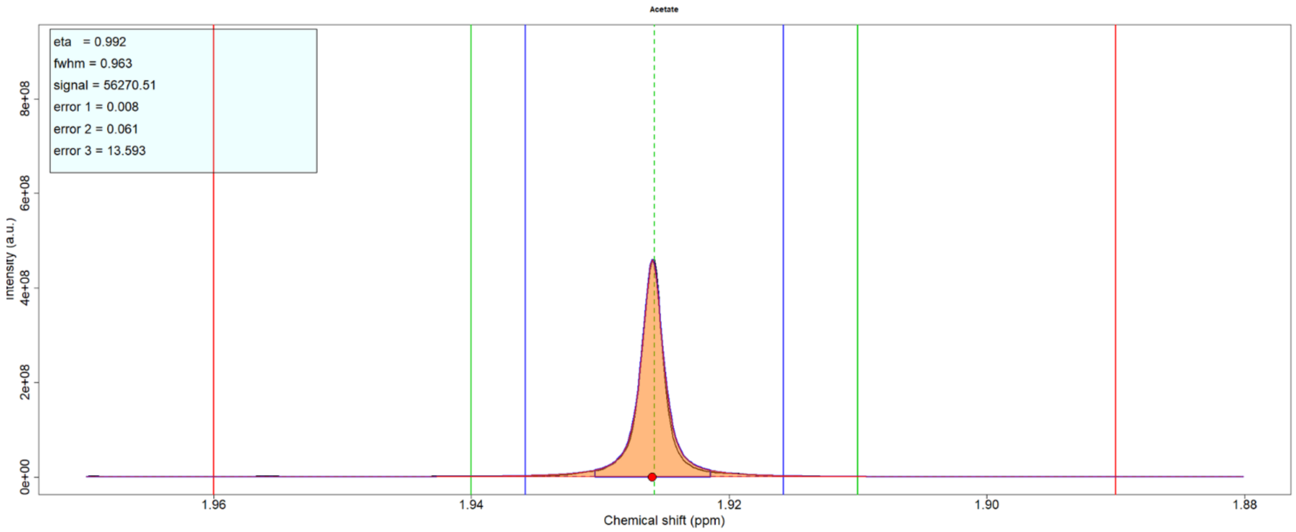
Acetate
eta = 0.992
fwhm = 0.963
signal = 56270.51
error 1 = 0.008
error 2 = 0.061
error 3 = 13.593
Intensity (a.u.)
Chemical shift (ppm)

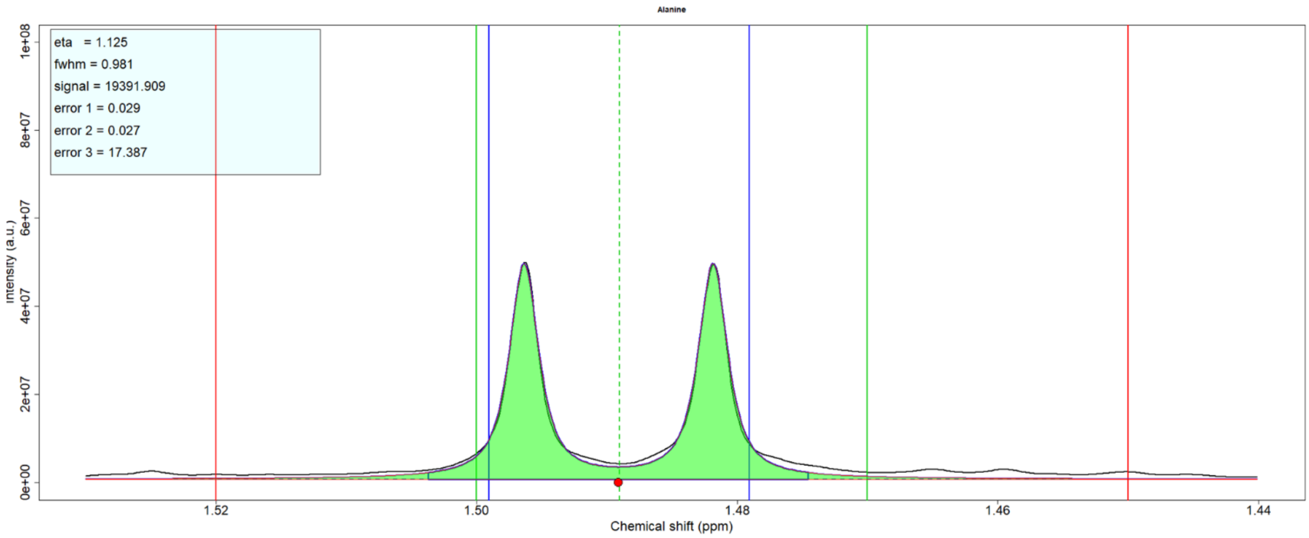
Alanine
eta = 1.125
fwhm = 0.981
signal = 19391.909
error 1 = 0.029
error 2 = 0.027
error 3 = 17.387
Intensity (a.u.)
Chemical shift (ppm)

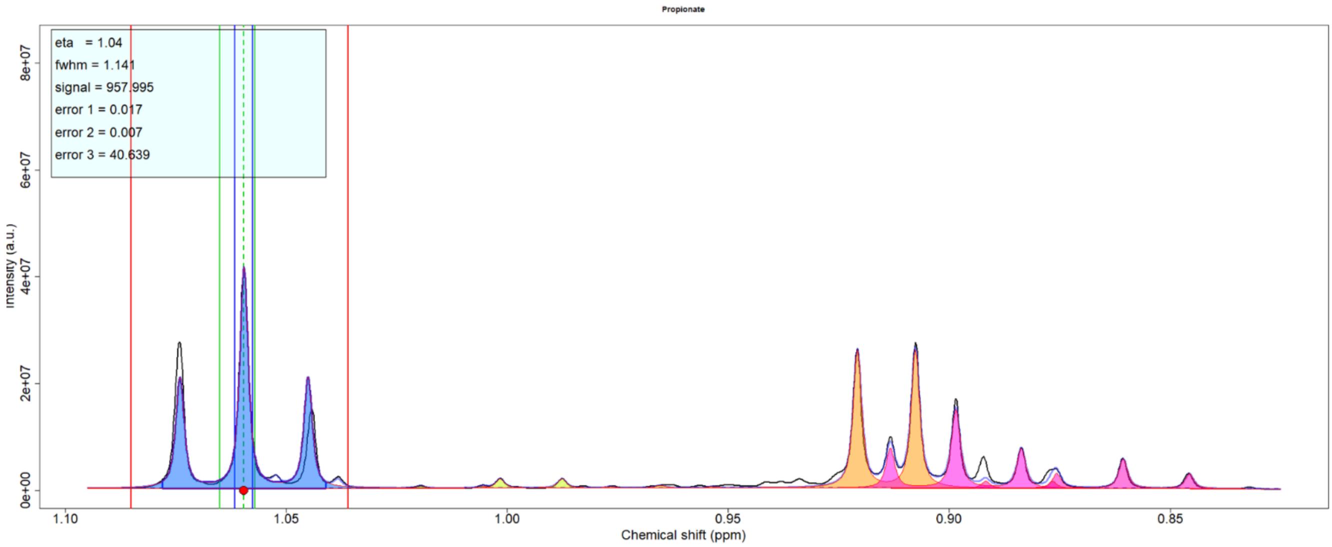
Propionate
eta = 1.04
fwhm = 1.141
signal = 957.995
error 1 = 0.017
error 2 = 0.007
error 3 = 40.639
Intensity (a.u.)
Chemical shift (ppm)

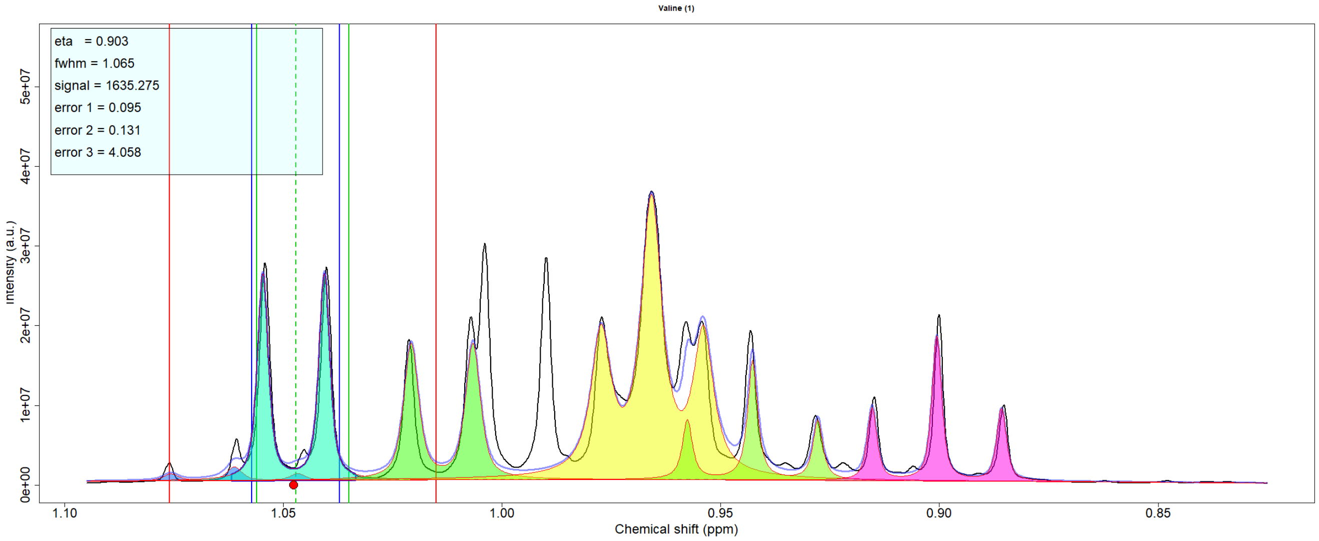
Valine (1)
eta = 0.903
fwhm = 1.065
signal = 1635.275
error 1 = 0.095
error 2 = 0.131
error 3 = 4.058
Intensity (a.u.)
Chemical shift (ppm)

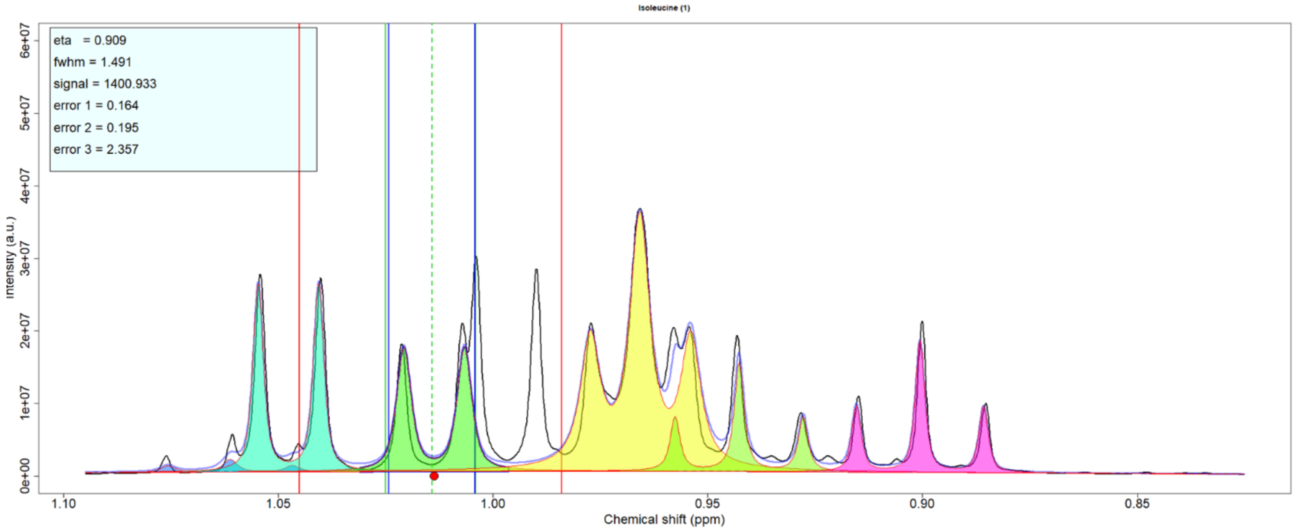
Isoleucine (1)
eta = 0.909
fwhm = 1.491
signal = 1400.933
error 1 = 0.164
error 2 = 0.195
error 3 = 2.357
Intensity (a.u.)
Chemical shift (ppm)

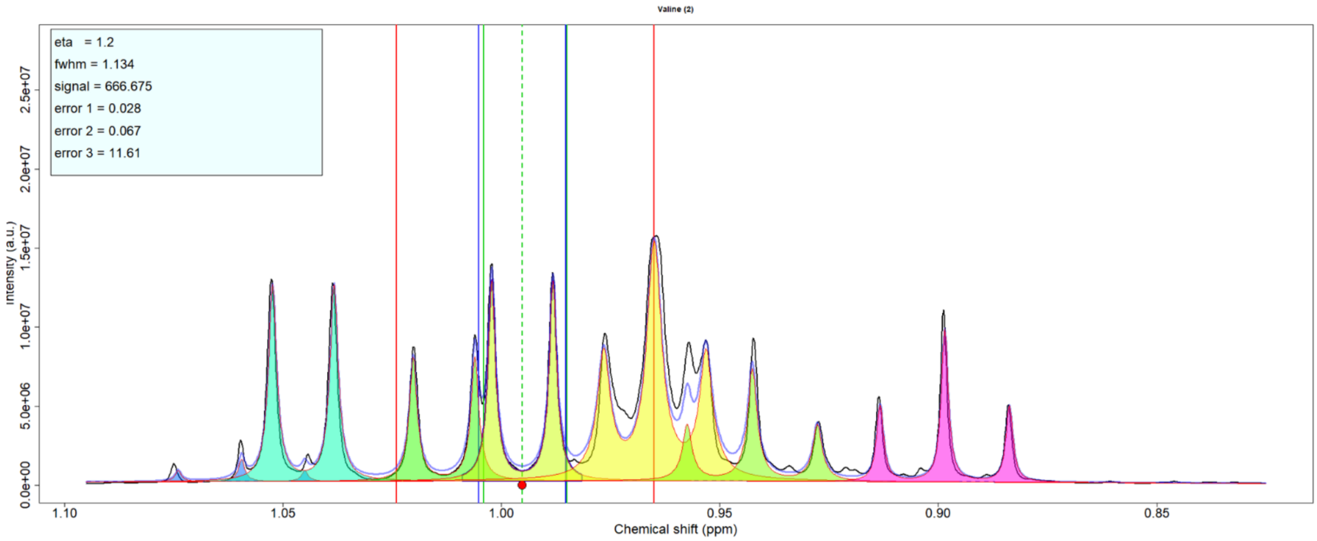
Valine (2)
eta = 1.2
fwhm = 1.134
signal = 666.675
error 1 = 0.028
error 2 = 0.067
error 3 = 11.61
intensity (a.u.)
0.0e+00
5.0e+06
1.0e+07
1.5e+07
2.0e+07
2.5e+07
1.10
1.05
1.00
0.95
0.90
0.85
Chemical shift (ppm)

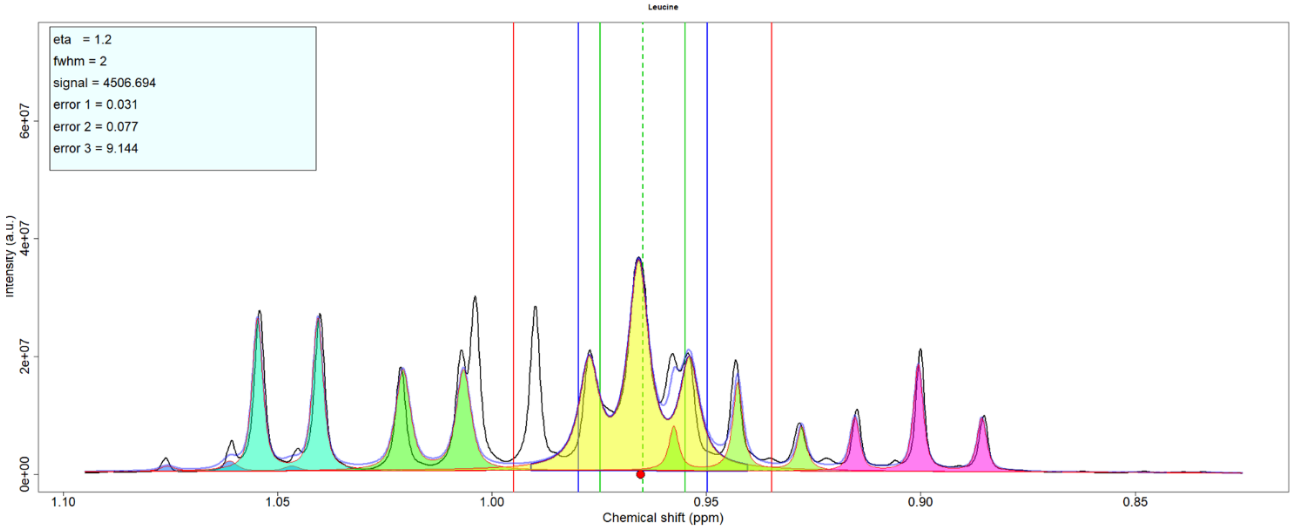
Leucine
eta = 1.2
fwhm = 2
signal = 4506.694
error 1 = 0.031
error 2 = 0.077
error 3 = 9.144
intensity (a.u.)
0e+00
2e+07
4e+07
6e+07
1.10
1.05
1.00
0.95
0.90
0.85
Chemical shift (ppm)

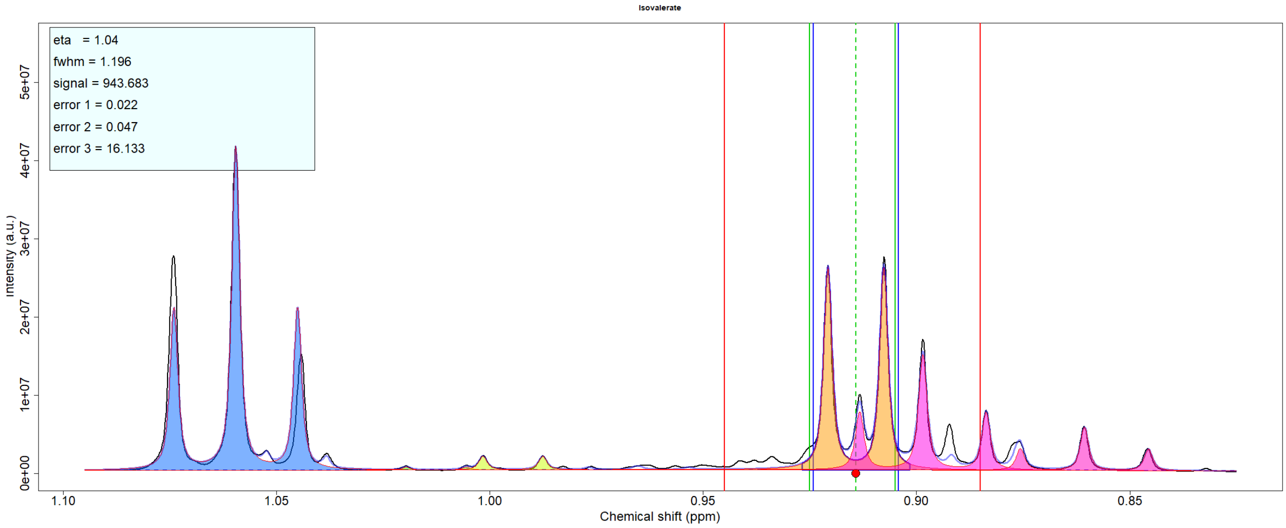
Isovalerate
eta = 1.04
fwhm = 1.196
signal = 943.683
error 1 = 0.022
error 2 = 0.047
error 3 = 16.133
intensity (a.u.)
0e+00
1e+07
2e+07
3e+07
4e+07
5e+07
1.10
1.05
1.00
0.95
0.90
0.85
Chemical shift (ppm)

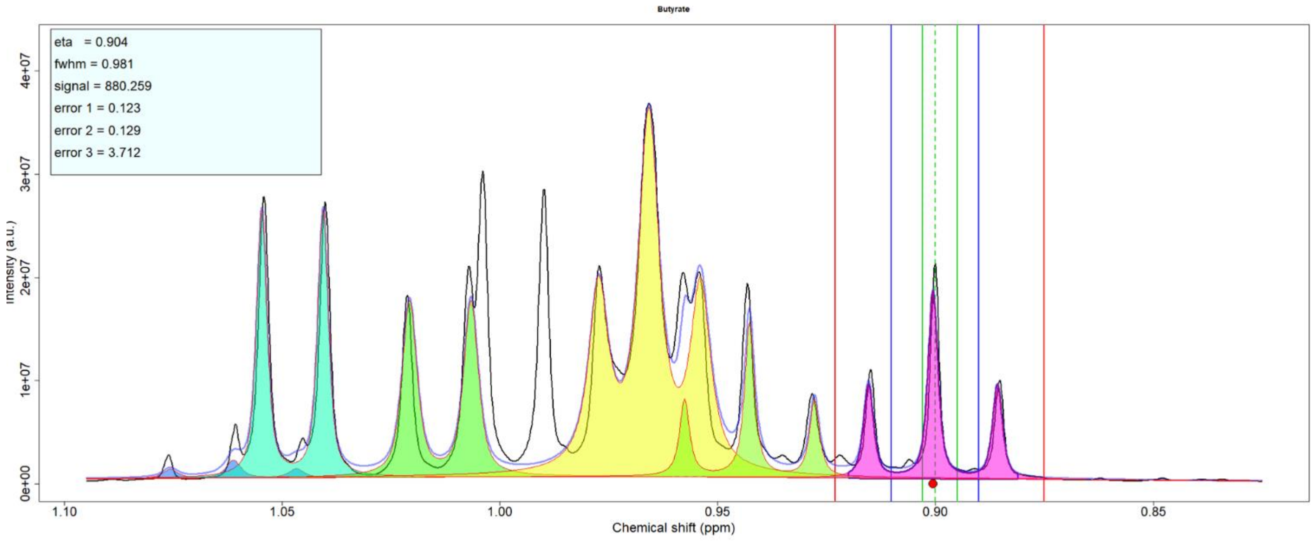
Butyrate
eta = 0.904
fwhm = 0.981
signal = 880.259
error 1 = 0.123
error 2 = 0.129
error 3 = 3.712
intensity (a.u.)
0e+00
1e+07
2e+07
3e+07
4e+07
1.10
1.05
1.00
0.95
0.90
0.85
Chemical shift (ppm)

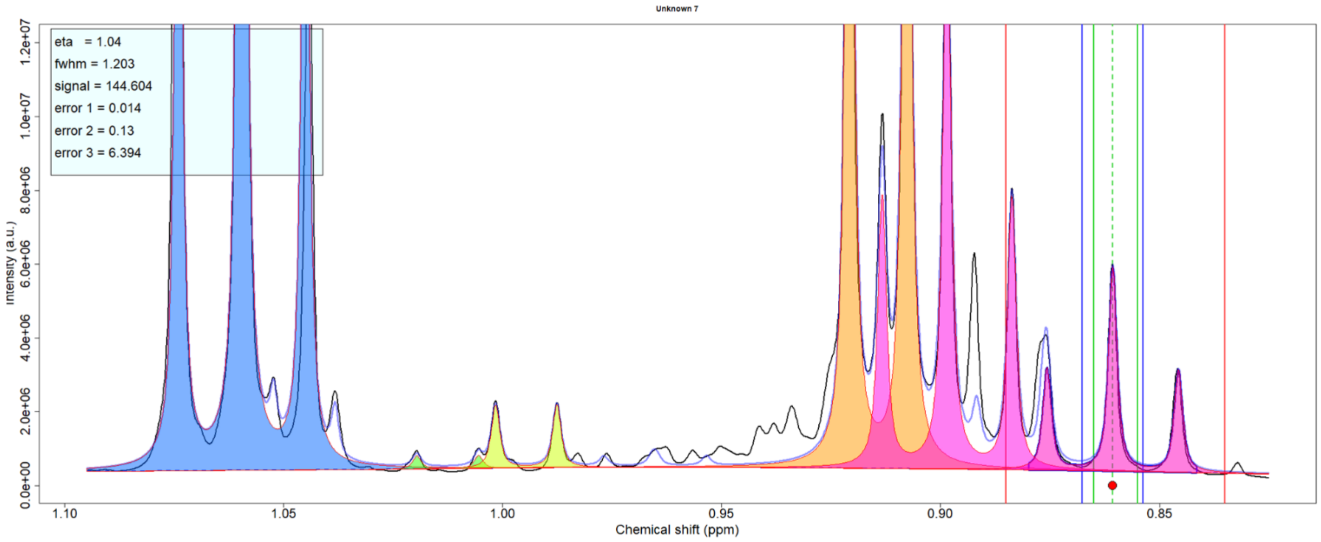
Unknown 7
eta = 1.04
fwhm = 1.203
signal = 144.604
error 1 = 0.014
error 2 = 0.13
error 3 = 6.394
intensity (a.u.)
0.0e+00
2.0e+06
4.0e+06
6.0e+06
8.0e+06
1.0e+07
1.2e+07
1.10
1.05
1.00
0.95
0.90
0.85
Chemical shift (ppm)

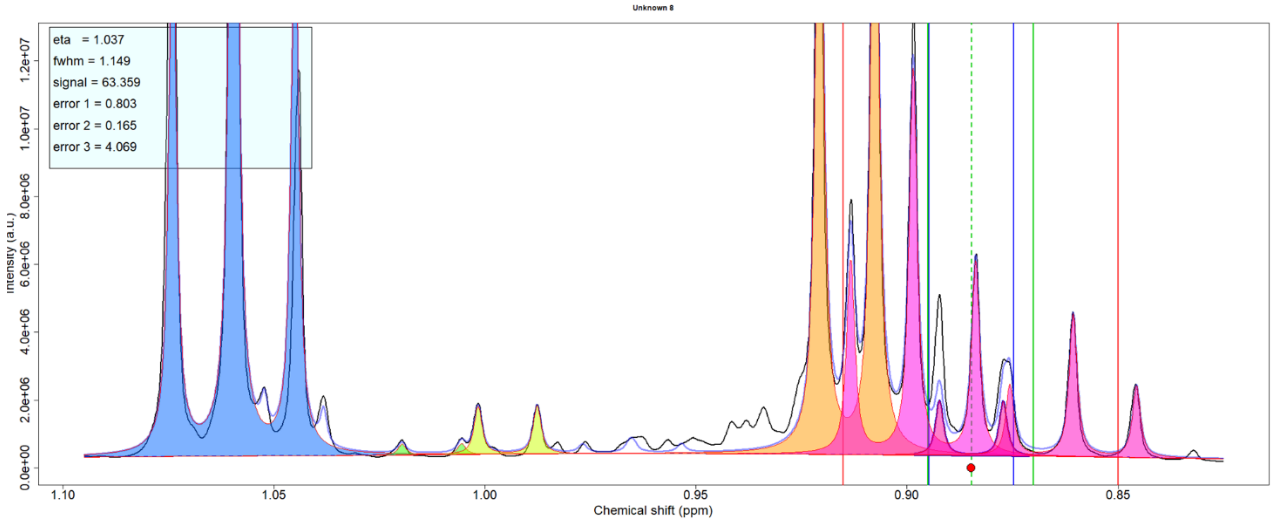
Unknown 8
eta = 1.037
fwhm = 1.149
signal = 63.359
error 1 = 0.803
error 2 = 0.165
error 3 = 4.069
intensity (a.u.)
0.0e+00
2.0e+06
4.0e+06
6.0e+06
8.0e+06
1.0e+07
1.2e+07
1.10
1.05
1.00
0.95
0.90
0.85
Chemical shift (ppm)
