# Supplementary material for: Metabolomics approach for predicting stomach and colon contents in dead Arctocephalus pusillus pusillus, Arctocephalus tropicalis, Lobodon carcinophaga and Ommatophoca rossii from sub-Antarctic region
Source: PLoS One. 2024 Apr 1;19(4):e0300319. doi: 10.1371/journal.pone.0300319 (PMC10984408; doi:10.1371/journal.pone.0300319)
Supplement: Attachment — Submitted filename: Response PLOS ONE.docx [file pone.0300319.s002.docx]

**Metabolomics approach for stomach and colon contents analysis in *Arctocephalus pusillus*, *Arctocephalus tropicalis*, *Lobodon carcinophaga* and *Ommatophoca rossii* from Sub-Antarctic region**

**TO** : PLOS ONE editor

**FROM** : Prof. Mkolo N.M.

Department: Biology & Environmental Science

Sefako Makgatho Health Sciences University

Medunsa, 0204

**January 2024**

**RE: RESPONSES TO PLOS ONE REVIEWERS**

**Prof Mkolo NM and the co-authors** submitted a manuscript titled “**Metabolomics approach for stomach and colon contents analysis in *Arctocephalus pusillus*, *Arctocephalus tropicalis*, *Lobodon carcinophaga* and *Ommatophoca rossii* from Sub-Antarctic region**” to the PLOS ONE for review.

The authors would like to thank the reviewers for their in-depth analysis and valuable comments. The committee recommended the following revision and besides them are the rebuttal thereof.

**Prof. NM Mkolo**

**Response to reviewers’ comments:**

**Rebuttal table**

|  | Reviewers’ comments | Response |
| --- | --- | --- |
| 1. | The aim of this review was to evaluate ‘Metabolomics approach for stomach and colon contents analysis in *Arctocephalus pusillus, Arctocephalus tropicalis, Lobodon carcinophaga and Ommatophoca rossii* from Sub-Antarctic region’. Thus, this manuscript represents a valid contribution to the understanding of the importance of using metabolomics technique for the examination of stomach and gut contents. Based on that, I consider the manuscript with interest to be published but with major revision. Nevertheless, before it can be published in its final form, I have some concerns that need to be addressed. There is repetition of sentences at some places and some of the sentences seem meaningless. Mostly old references have been cited. So, overall the manuscript needs an exhaustive revision before it can be considered for publication. | Thank you for the comment exhaustive revision have been done in paper. Sentences which are repeated were removed. Moreover, old references were noted and revised. |
| 2. | **Title:** The title does not convey the significance or implications of the study's findings and making it difficult to grasp the focus of the study. | The title was corrected |
| 3. | **Abstract:** Abstract may be unclear to readers without a specific background in the subject. It is of concern that the abstract could have been catchier about the importance of study. Overall, the results aren’t reflected in a comprehensive manner and don’t signify the importance of study. **Contributions to the field:** The contributions to the field statement could be improved. I suggest that this section be rewritten. Moreover, authors didn’t provide the choice of selection of the species. I think it is a good idea to conclude by summarizing the practical implications of the study's findings. | The abstract was rewritten and content on importance of the study and contribution to the field was added. |
| 4. | **Introduction:** The introduction is quite short (for the information it aims to provide) and could benefit from being more elaborative providing the significance of study in reference to relevant literature. Most significantly, the introduction also lacks a clear thesis statement that succinctly summarizes the main objectives and purpose of the study. | The introduction was corrected and more information was provided. |
| 5. | **Materials and Methods:** I find this section to be generally well-written. It provides sufficient details on the study area, sampling, identification etc. | We thank you for the comment. |
| 6. | **Results:** I find the results section to be comprehensive and detailed. | We thank you for the comment. |
| 7. | **Discussion and conclusion:** Again, I find this section to be comprehensive and detailed. But some sentences are unnecessarily long and complex, which makes the text difficult to read; break them up for clarity. Old references must be replaced with latest ones. Conclusion hasn’t been well synthesized lacking coherence with the focus and findings of the study. | We thank you for the comment and this section was corrected accordingly. |
